# Noise suppression beyond the thermal limit with nanotransistor biosensors

**DOI:** 10.1038/s41598-020-69493-y

**Published:** 2020-07-29

**Authors:** Yurii Kutovyi, Ignacio Madrid, Ihor Zadorozhnyi, Nazarii Boichuk, Soo Hyeon Kim, Teruo Fujii, Laurent Jalabert, Andreas Offenhaeusser, Svetlana Vitusevich, Nicolas Clément

**Affiliations:** 1 Bioelectronics (IBI-3), Forschungszentrum Jülich, 52425 Jülich, Germany; 2 LIMMS-CNRS/IIS, Institute of Industrial Science, The University of Tokyo, Tokyo, 153–8505 Japan

**Keywords:** Biophysics, Biotechnology, Nanoscience and technology, Physics

## Abstract

Transistor biosensors are mass-fabrication-compatible devices of interest for point of care diagnosis as well as molecular interaction studies. While the actual transistor gates in processors reach the sub-10 nm range for optimum integration and power consumption, studies on design rules for the signal-to-noise ratio (S/N) optimization in transistor-based biosensors have been so far restricted to 1 µm^2^ device gate area, a range where the discrete nature of the defects can be neglected. In this study, which combines experiments and theoretical analysis at both numerical and analytical levels, we extend such investigation to the nanometer range and highlight the effect of doping type as well as the noise suppression opportunities offered at this scale. In particular, we show that, when a single trap is active near the conductive channel, the noise can be suppressed even beyond the thermal limit by monitoring the trap occupancy probability in an approach analog to the stochastic resonance effect used in biological systems.

## Introduction

**Figure 1:**
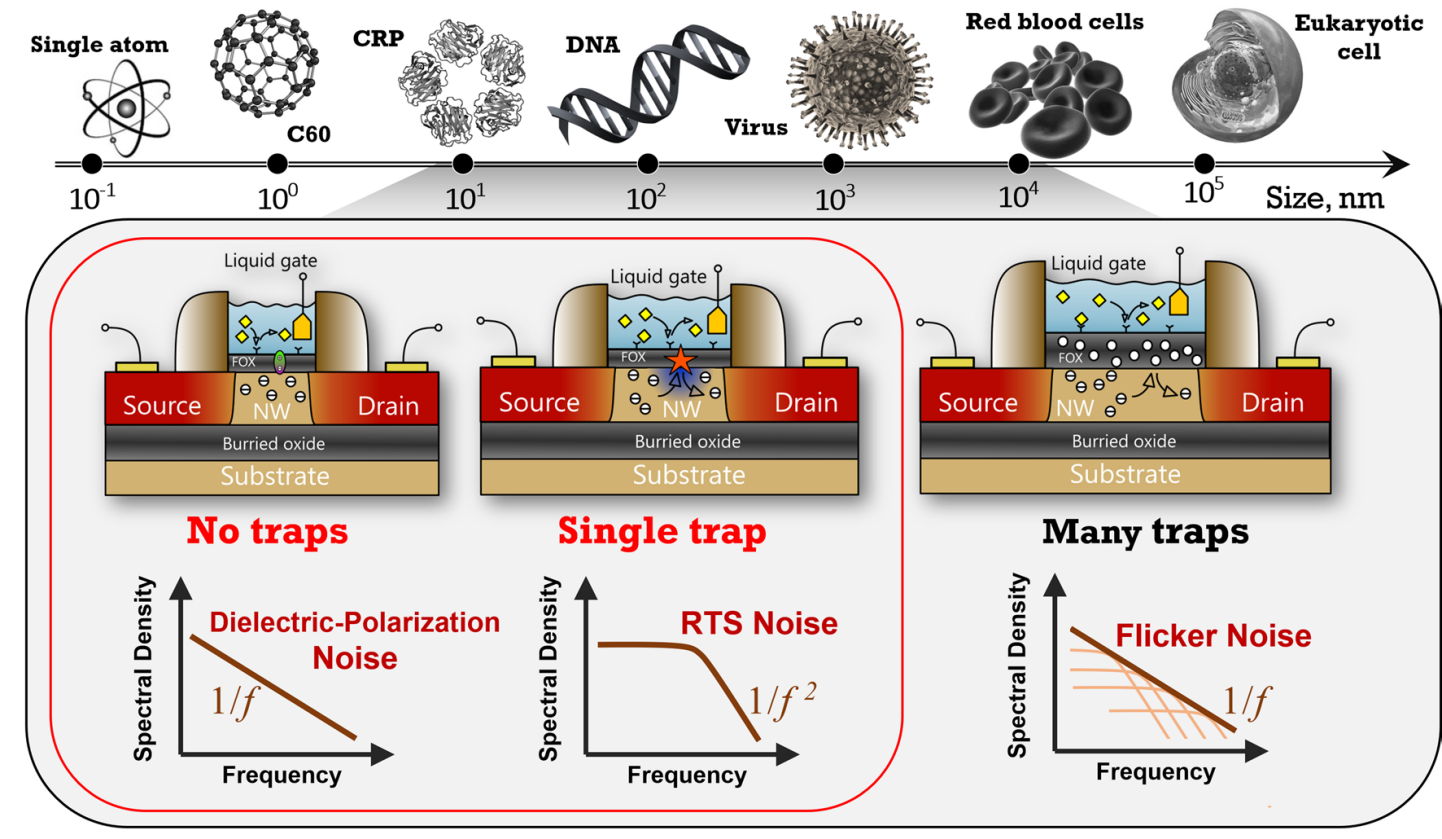
Schematic illustration of the dimensions of transistor-based biosensors compared to typical biological systems, as well as the impact of such dimensions on the sources of noise in such devices.

Transistor-based biosensors are now widely used as integrated semiconductor devices for genome sequencing^1^. They are still being further integrated for statistical study^1,2^, pico- or nano-liter volume analysis^3–5^ or single-molecule sensing^6,7^. Smallest nanotransistor-based biosensors are very similar to mass-production state-of-the-art semiconductor transistors^8,9^, have dimensions close to small biological objects (see Fig. 1), and tend to have a very low charge noise ${S}_{q}$. In particular, the sub-elementary charge or single-charge sensitivity ability in liquid^10^ is promising for the development of the non-optical version of single-molecule digital nanoarrays^11^, or nanoelectrochemistry^12^. In contrast, larger devices, whose dimensions are typically similar to a biological cell (see Fig. 1), have a larger charge noise but tend to have a very low input-referred voltage noise ${S}_{{V}_{G}}$. Several studies have recently investigated quantitatively the role of gate area $A$ for ${S}_{{V}_{G}}$ noise^13,14^ in the range $A>1$ µm^2^. In principle, ${S}_{{V}_{G}}$ reflects the smallest change in analyte concentration that can be detected with such biosensors. The simplest and effective model for noise (see Eqs. (1a) and (1b)) is based on the fluctuation of the number of active defects (typically gate oxide traps)^15^.

1a $${S}_{q}=\frac{{q}^{2}{N}_{ot} WL}{f}=\frac{{{q}^{2}N}_{ot} A}{f}$$

1b $${S}_{{V}_{G}}=\frac{{S}_{I}}{{g}_{m}^{2}}=\frac{{S}_{q}}{{(C}_{G} WL)^{2}}=\frac{q^{2}{ N}_{ot} }{{C}_{G}^{2} A f}$$

where ${S}_{I}$ is the power spectral density of current noise, ${g}_{m}$ is the transconductance, $q$ is the elementary charge, ${N}_{ot}$ is the density of oxide traps (or other charge trapping source), ${C}_{G}$ is the gate oxide capacitance per surface unit, and $f$ is the frequency.

It appears from Eqs. (1a) and (1b) that ${S}_{q}$ and ${S}_{{V}_{G}}$ scale as $A$ and ${A}^{-1}$, respectively. Equations (1a) and (1b) assume that ${N}_{ot} A\gg 1$, and that the interaction of chemical species with the sensor itself is the signal, therefore it does not affect ${S}_{{V}_{G}}$_._ This latter assumption seems to be valid in the majority of cases^5,10,16^ if we neglect drift effects affecting the very low-frequency signal^17,18^. Considering a typical lower range of ${N}_{ot}=1{0}^{8} c{m}^{-2}$ for the state-of-the-art devices, we see that ${N}_{ot} A=1$ corresponds to $A=1 {\mu m}^{2}$ as a lower boundary^13–15^, and therefore, one can wonder what is the optimal design rule when $A<1 {\mu m}^{2}$. Even more interestingly, nanoscale devices can offer unique opportunities for noise suppression due to the absence of traps^10^, correlation effects^19,20^, and single-trap phenomena^21–23^. Here, we address both questions and show in particular that, by analogy with the stochastic resonance (SR) noise suppression approach found in biological systems, the exploitation of single-trap phenomena in nanoscale devices can be quantitatively described and used for noise suppression, even beyond the thermal noise limit^2,24^.

## Scaling effect on charge and voltage noise and experimental results

**Figure 2:**
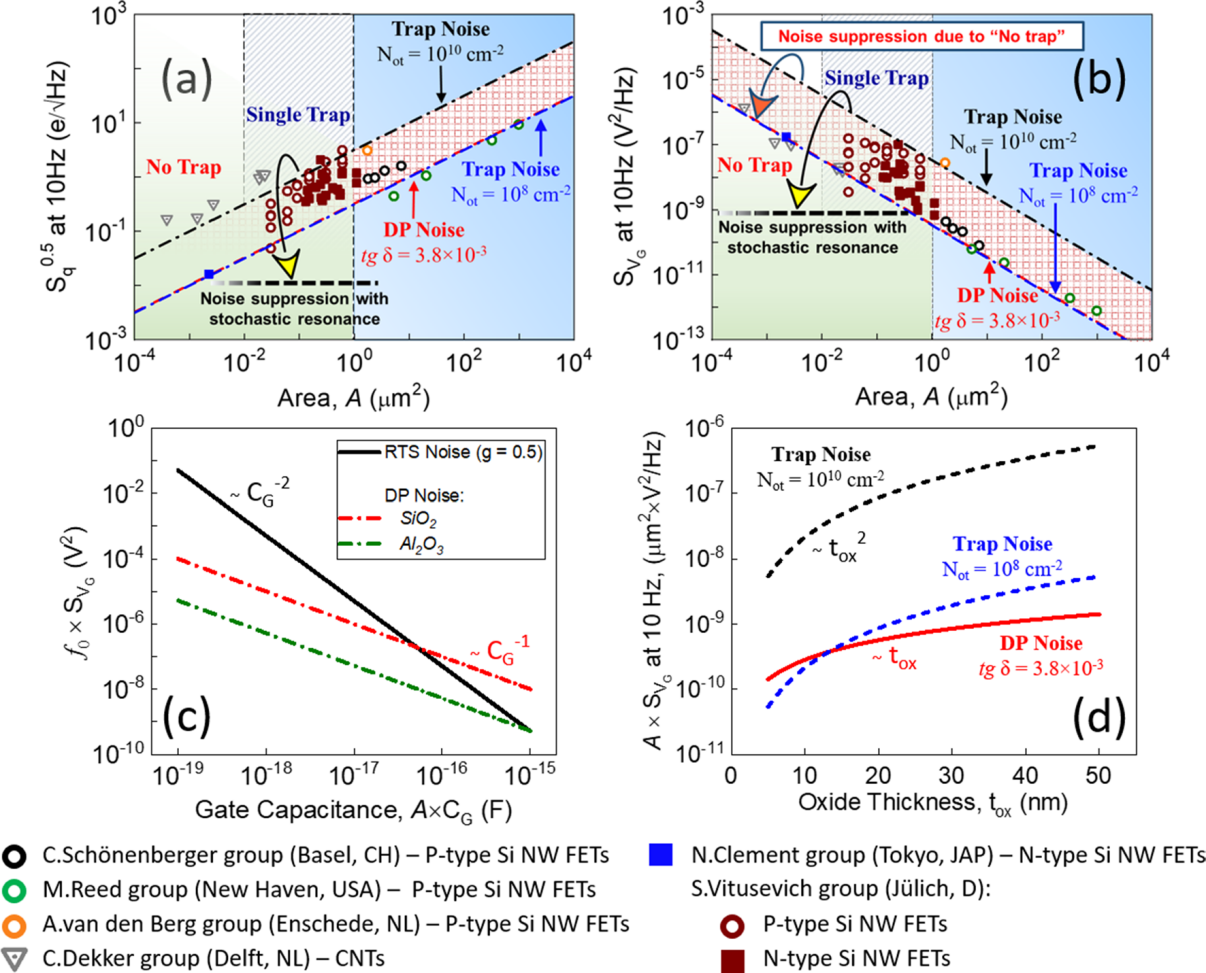
Noise in nanoscale biosensors. Experimental results of (**a**) ${S}_{q}^{ 0.5}$ and (**b**) ${S}_{{V}_{G}}$ taken at 10 Hz as a function of gate area $A$ obtained by different research groups: C. Schönenberger^13^, M. Reed^28^, A. van den Berg^14^, C. Dekker^26^, N. Clement^10^, S. Vitusevich (see SI). The dashed horizontal lines illustrate the notion of noise suppression indicating the noise level related to the results shown in Fig. 5(d). Scaling trend of ${S}_{{V}_{G}}$ noise on (**c**) gate capacitance and (**d**) oxide thickness calculated for different conditions indicated in the figure.

Figure 2a,b show some of the state-of-the-art experimental results of charge noise (${S}_{q}^{ 0.5}$) and input-referred voltage noise ${S}_{{V}_{G}}$ as a function of $A$, with $A$ down to a few hundred ${nm}^{2}$, including some additional experimental data obtained for both N and P-type devices in the range $A<1 {\mu m}^{2}$ (see Supplementary Information (SI) for the details). Rather counterintuitively, we see that guidelines based on Eqs. (1a) and (1b) can be considered as reasonable approximations over the full range of $A$, even in the absence of traps. A way to understand this is to introduce the “charge noise”, that simply considers ${S}_{q}$ as a constant in the whole gate bias range. It was initially introduced by the mesoscopic physics community to describe noise in elementary charge-sensitive electrometers (whose origin was typically attributed to fluctuating charges in the substrate)^25^, but also used by the biosensors community to explain noise in nanoscale silicon transistors^10,13^, carbon nanotubes^26^, graphene FETs^27^ or PEDOT:PSS-based organic electrochemical transistor devices^16^.

A quantitative attempt to the charge noise came from the measurement of noise in trap-free devices, and the origin was attributed to the dielectric polarization (DP) noise related to the thermal fluctuation of dipoles in the gate oxide^10^. The consideration of a typical dielectric loss tangent *tg δ* = 3.8 × 10^–3^ for the SiO_2_^10,29^ gate dielectric could provide a quantitative description as follows:

2a $${S}_{q}=\frac{2kT tg\delta {C}_{G} A}{\pi f}$$

2b $${S}_{{V}_{G}}=\frac{2kT tg\delta }{\pi {C}_{G} A f}$$

where $k$ is the Boltzmann constant and $T$ is the temperature.

Interestingly, we stress that considering a double-layer capacitance of 0.2 F m^-2^ and *tg* *δ* = 5 × 10^–3^ Eq. (2a) could also provide a quantitative agreement to the charge noise measured for liquid-gated carbon nanotube transistor sensors^26^ (see Fig. 2a).

## Noise suppression in nanoscale devices

Nanoscale devices offer unique opportunities for noise suppression. In this section, we propose a critical review of the various approaches and present the results for both N and P-type nanodevices.

### P-type and N-type sub-µm devices

As it is predicted by Eq. (1a), ${S}_{{V}_{G}}$ noise is inversely proportional to the gate area for both N-type and P-type FETs. However, it should be noted that the authors in Ref.^15^ suggested that P-type transistors might have a lower noise level than N-type devices due to lower ${N}_{ot}$ for P-type structures in relation to different tunneling parameters (e.g. carriers effective mass) for electrons and heavy holes. Our experiments performed with nanoscale devices fabricated in the same technological run show that this is not necessarily the case for sub-µm devices (see Fig. 2a,b). One reason could be that the energy distribution of the few traps in scaled devices is pretty similar for both N- and P-type structures performed with the same fabrication protocol. Another one would be that the Coulomb repulsion effect between traps could be more effective for P-type devices. Such an effect is seen only when a transistor has multiple traps^19^ (e.g. typically for micrometric devices). In the case of a single trap, ${S}_{{V}_{G}}$ noise increases by up to two orders of magnitude when compared to no trap for both N-type and P-type devices due to a random-telegraph signal (RTS) noise whose amplitude $\Delta I={g}_{m}\times {q}^{*}/({C}_{G}\times A)$, where ${q}^{*}$ being an effective charge of about $0.5 q$ for SiO_2_ that accounts for image charge effects^19^. RTS noise has a Lorentzian power spectrum shape (see Fig. 1) that can be evaluated as^19^:

3 $${S}_{{V}_{G}}=\frac{4g {(1-g)}^{2} {\tau }_{e} {({q}^{*}/{C}_{G})}^{2}}{1+{(2\pi \left(1-g\right){ \tau }_{e} f)}^{2}}$$

where $g$ denotes the trap occupancy probability (g-factor) given by:

4 $$g\left(t\right)=\frac{{\tau }_{e}}{{\tau }_{e}+{\tau }_{c}}$$

where ${\tau }_{e}$ is the emission time of a charge from the trap and ${\tau }_{c}$ is the capture time in the trap. RTS noise is maximized relative to the background DP noise at the frequency equal to the Lorentzian corner frequency ${f}_{0}$. By considering the probability of the trap to be occupied equal to 50%, the maximum of RTS noise can be estimated as^19^:

5 $${S}_{{V}_{G}}(RTS)\approx 0.08\times \frac{{(\frac{{q}^{*}}{{C}_{G}\times A})}^{2}}{{f}_{0}}$$

According to Eq. (5), RTS noise tends to increase with capacitance decrease showing a stronger dependence than DP noise. RTS noise is typically above DP noise (see Fig. 2b,c). Such behavior demonstrates the effect of the presence of a single trap on the nanotransistor biosensor performance. However, as we discuss below, RTS noise can be suppressed by considering the single-carrier trapping-detrapping process as a signal rather than a parasitic effect. Moreover, better performance of nanobiosensors exploiting RTS is expected for devices covered with high-k dielectrics. Typically, high-k materials possess higher dielectric constants and lower values of dielectric loss tangent compared to the conventional SiO_2_. This leads to larger RTS amplitude and lower DP noise and, therefore, the improved performance of single-trap phenomena is expected in nanotransistors with high-k gate insulators.

### Dual-gate devices

The use of dual-gates (gate coupling effect^2,22^) for the nanotransistor biosensors in which a liquid-gate remains fixed and a back-gate is monitored has attracted substantial interest due to the possibility to capacitively amplify the signal by the ratio of the top gate to the bottom gate capacitances^30^. This approach is, however, not necessarily providing a larger signal-to-noise ratio (S/N) as the noise is amplified exactly in the same manner. Still, one can argue that this approach provides noise-free amplification, which can simplify the electronics acquisition setup.

### Defect-free devices

The second noise suppression effect due to the “nanometer dimension” is the fact that there are statistically no oxide traps for devices of a few tens of nanometers (see Fig. 2b). The gain compared to devices with traps (at fixed capacitance) is about a factor 12 (1200%)^19^. One could have expected a gain of several orders of magnitude in ${S}_{{V}_{G}}$, but it is restricted due to the presence of the thermal DP noise (see Fig. 2b,d). As Eqs. (1b) and (2b) have different dependence on ${C}_{G}$, ${S}_{{V}_{G}}$ is relatively lower for DP noise with thicker oxides when compared to the trapping/detrapping noise (see Fig. 2d).

### Single-trap phenomena as a stochastic resonance effect

**Figure 3:**
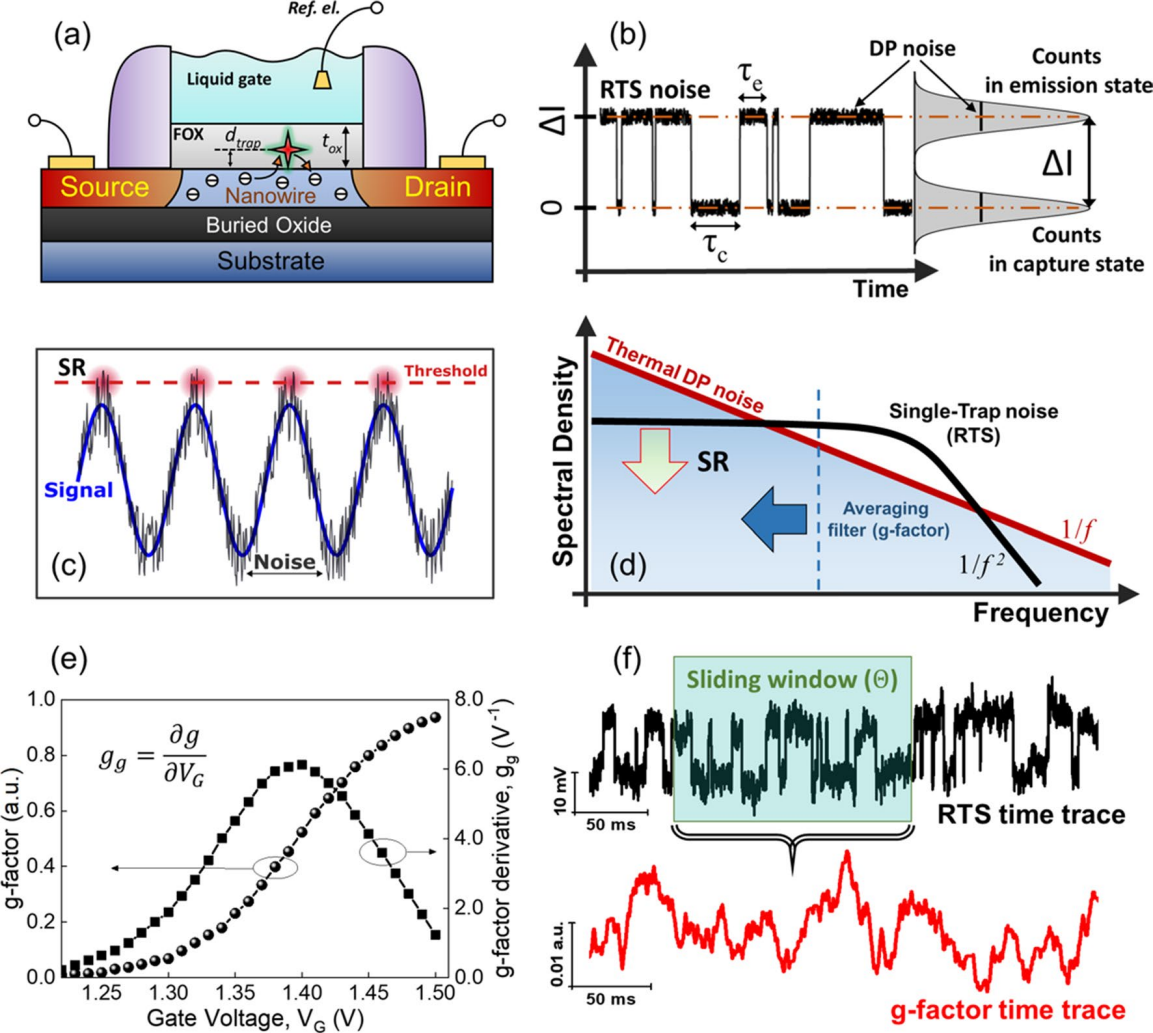
Single-trap phenomena in nanoscale biosensors. (**a**) Schematic illustration of a liquid-gated Si NW FET with a single trap that induces (**b**) two-level RTS fluctuations of the drain current. (**c**) Schematic interpretation of SR: an optimal amount of white noise is added to a system to detect weak signals under the system threshold. (**d**) DP noise suppression due to single-trap phenomena considering a single trap as a nonlinear bistable system that can amplify the signal in the regime of SR. (**e**) Trap occupancy probability (g-factor) and its derivative plotted as a function of gate voltage for simulated RTS noise. (**f**) Schematic illustration of the conversion of RTS voltage fluctuations into the fluctuations of trap occupancy probability (g-factor noise).

The third noise suppression effect, as introduced in^21,22^, aims to exploit the presence of a single active trap in a gate dielectric layer of a nanotransistor, where RTS noise is observed (see Fig. 3a,b). Such an RTS effect is usually avoided as it increases the noise level (see Fig. 2b,c), but if RTS parameters (i.e. trap occupancy probability, time constants) are monitored (see Fig. 3b), then RTS noise becomes a signal. Intuitively, one could expect that the use of RTS as a signal would provide a gain corresponding to the difference between a single-trap and a trap-free device, e.g. between one and two orders of magnitude (see Fig. 2a,b). Below, we show that the potential of single-trap phenomena for the noise suppression is even larger and that it is similar to the SR effect observed in biology^31^, enabling here to overpass the thermal DP noise limit. The idea beyond this is that the addition of white noise to a signal that is non-measurable below a given threshold can become measurable (see Fig. 3c). As RTS is nothing but a white noise below a cut-off frequency that is added to the signal of interest, there are obviously some similarities (Fig. 3d). However, a technical difference comes from the fact that RTS time constants are related to the signal of interest (surface potential), which is usually not the case for the white noise. Below, we combine theory and experiments to push the limits of noise suppression with single-trap phenomena.

## Noise suppression beyond the thermal limit

In this section, the aim is to propose a theoretical framework for the signal-to-noise ratio in the case of the single-trap phenomena approach. We consider the trap occupancy probability as the signal and evaluate the noise of $g$ to determine the S/N ratio (see Fig. 3e,f). We demonstrate experimentally, numerically, and analytically that under optimized conditions, the S/N ratio can be beyond that of the thermal noise in trap-free devices.

### Trap occupancy probability ${\varvec{g}}$ and numerical simulation

The usual signal in transistor-based biosensors is a shift of drain current, and current fluctuations are noise. In contrast, we define the signal in single-trap-based biosensors^21,22,32^ as trap occupancy probability $g$. To calculate the g-factor noise (fluctuations in time) considering two-level RTS time trace, one can extract $g\left(t\right)$ over a given window $\Theta$ directly from the distribution of the voltage fluctuations (see Fig. 3f). Then, by sliding the window along with the RTS time trace one can obtain a new time trace with the trap occupancy factor fluctuations in time. The time-domain g-factor data can be then translated into frequency spectrum resulting in the power spectral density ${S}_{g}$.

### Experimental results

**Figure 4:**
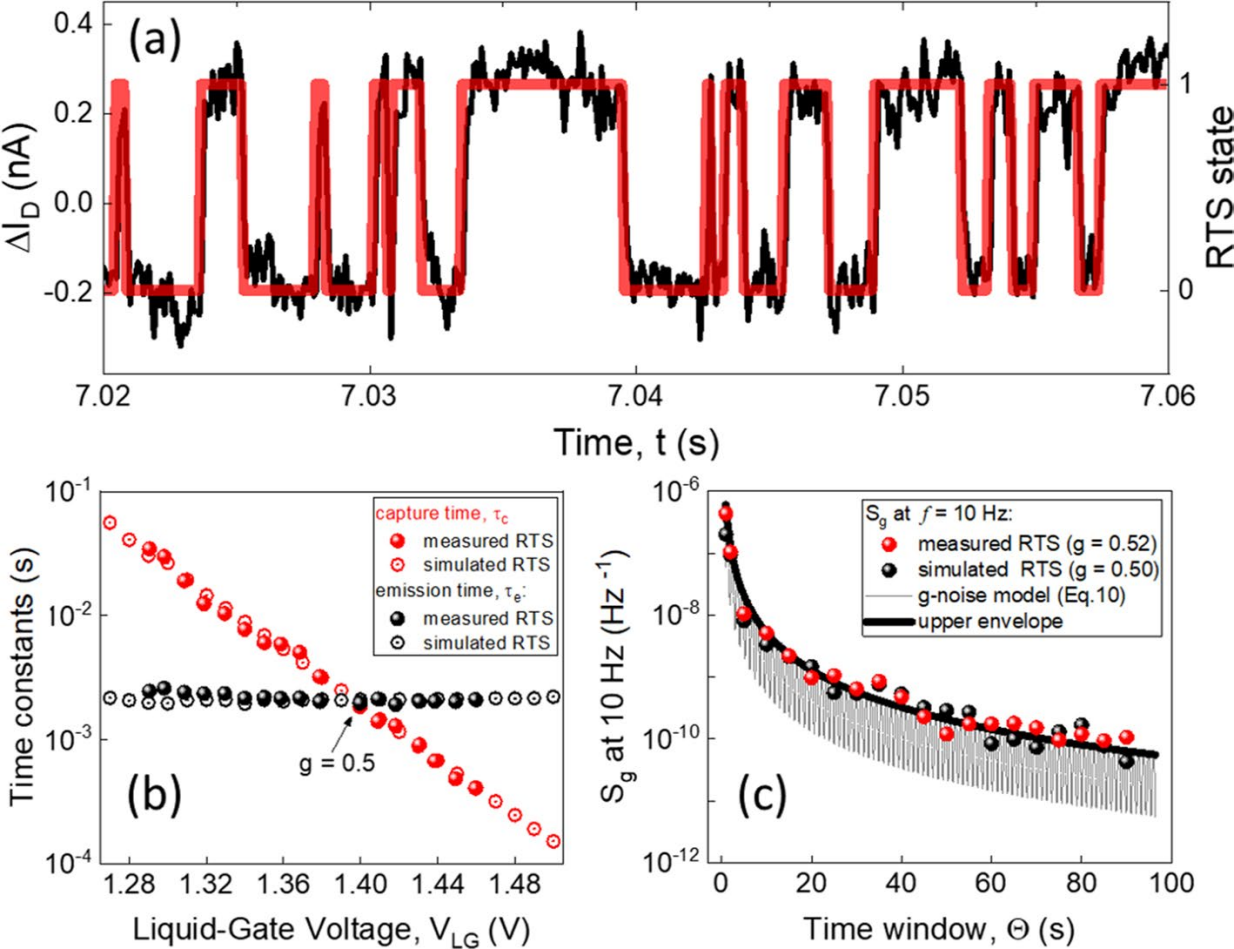
RTS noise characteristics. (**a**) A two-level drain current RTS fluctuations and the corresponding extracted RTS states measured in the 100 nm wide and 100 nm long liquid-gated Si NW FET. (**b**) Capture and emission time constants vs. liquid-gate voltage extracted for simulated and measured RTS time traces. (**c**) ${S}_{g}$ noise at 10 Hz vs. time window calculated for different conditions.

Figure 4a shows the two-level drain current fluctuations measured for the 100 nm wide and 100 nm long liquid-gated Si NW FET. The device was fabricated using a previously reported protocol^32^. A brief description of the main fabrication steps is also presented in Supporting information (SI) of this work. All noise measurements were performed in a custom-built Faraday cage using a fully-automated ultralow-noise measurement setup^22,33^. The transistor demonstrating RTS noise behavior was biased in the linear operation regime and measured at room temperature. To extract drain current states for measured RTS time trace a method based on a hidden Markov model^34,35^ was applied (see Fig. 4a). Average capture and emission time constants characterizing measured RTS process are shown in Fig. 4b. The average emission characteristic time remains about constant, while the average capture characteristic time demonstrates a strong dependence on the liquid-gate voltage applied. It should be noted that such behavior of RTS time constants is typical for liquid-gated nanowire-based FET devices^22,32,36^.

In order not to be limited statistically and to have long enough RTS time traces for calculation of g-factor noise, we also generated RTS noise numerically using master Equations^37^ (see Equations S1-S3 in SI) with additional consideration of DP noise^10^. Simulated RTS noise has characteristics similar to that obtained for experimentally measured RTS noise as it is shown in Fig. 4b. The trap occupancy factor noise taken at 10 Hz for both measured and simulated time traces is plotted in Fig. 4c. It should be noted that the data shown in Fig. 4c is obtained for RTS with $g=0.5$, which corresponds to the case when the trap energy level coincides with the Fermi level of the system. At this condition the number of transition events between the states is maximized, so the noise introduced by the calculation of the trap occupancy factor (g-factor noise) is also maximized.

As can be seen from Fig. 4c, the g-factor noise decreases with increasing the time window $\Theta$. The dependence of g-factor noise against the time window can be explained by considering the fact that the larger time window contains more transition events enabling g-factor to be estimated with higher accuracy, as illustrated in Fig. 3f.

### g-factor noise analytical model

Let’s consider a two-level RTS signal ${X}_{t}$ that jumps between states 0 and 1. The transition probabilities $P$ for an RTS with states (0, 1) and rates $(\lambda ,\mu )$ to jump from states 0 to 1, and 1 to 0, respectively, are given by Kolmogorov´s forward equation:

6 $${P\left(t\right)=\left[{\mathbb{P}}\left({X}_{t}=j|{X}_{0}=i\right)\right]}_{i,j}=\frac{1}{\lambda +\mu }\left(\begin{array}{cc}\mu & \lambda \\ \mu & \lambda \end{array}\right)-\frac{1}{\lambda +\mu }{e}^{-\left(\lambda +\mu \right)t}\left(\begin{array}{cc}-\lambda & \lambda \\ \mu & -\mu \end{array}\right)$$

Then, we consider the trap occupancy probability $g$ (our signal), averaged over a time window $\Theta$ and defined as:

7 $${g}^{\Theta }\left(t\right):=\frac{1}{\Theta }{\int }_{t}^{t+\Theta }{1}_{\left\{{X}_{S}=1\right\}}ds$$

where ${1}_{\left\{{X}_{S}=1\right\}}$ is the indicator function (equal to 1 if ${X}_{S}=1$). To obtain the autocorrelation function $C\left(s\right)$ of $g$, we consider the expected value $E$ and obtain:

8 $$C\left(s\right)=E\left[g\left(t\right)g\left(t+s\right)\right]=E\left[\left(\frac{1}{\Theta }{\int }_{t}^{t+\Theta }{1}_{\left\{{X}_{s}=1\right\}}ds\right)\times \left(\frac{1}{\Theta }{\int }_{t+s}^{t+s+\Theta }{1}_{\left\{{X}_{s}=1\right\}}ds\right)\right]$$

where $s$ is the time lag. Then, $C\left(s\right)$ can be written as:

9 $$C\left(s\right)=\frac{1}{{\Theta }^{2}}\left[\left({\int }_{0}^{\Theta }\underset{s}{\overset{s+\Theta }{\int }}{{\mathbb{P}}(X}_{max\left(u, v\right)}=1 \left| {X}_{min\left(u, v\right)}=1\right)\times {{\mathbb{P}}(X}_{min\left(u, v\right)}=1)du dv\right)\right]$$

We see that the autocorrelation function follows two regimes that are related to the averaging filter and the stochastic charge transfer, respectively (see Fig.S5). After some simplifications, an analytical model can be obtained for the power spectral density of $g$, in the case where $\lambda =\mu =\gamma$ (i.e. $g=0.5$) as:

10 $${S}_{g}^{\left(\Theta \right)}(\omega )=\frac{2\gamma \left(1-\cos\left(\Theta \omega \right)\right)}{\Theta^{2} \omega^{2} \left(4{\gamma }^{2}+\omega^{2} \right)}$$

where $\omega =2\pi f$ and $\Theta$ is a duration of a sliding time window (see Fig. 3(f)).

### Input-referred g-factor noise ${{\varvec{S}}}_{{\varvec{g}}{\varvec{g}}}$

To compare the performance and efficiency of the nanotransistor sensors exploiting single-trap phenomena, one should first introduce and calculate an equivalent input-referred noise caused by the variation of the g-factor. This can be done similarly as for the voltage noise (see Eq. (1b)) defining the input-referred trap occupancy factor noise as:

11 $${S}_{gg}=\frac{{S}_{g}}{{g}_{g}^{ 2}}$$

where ${S}_{g}$ is the g-factor power spectral density and ${g}_{g}$ is the g-factor derivative calculated as $\frac{\partial g}{{\partial V}_{G}}$.

**Figure 5:**
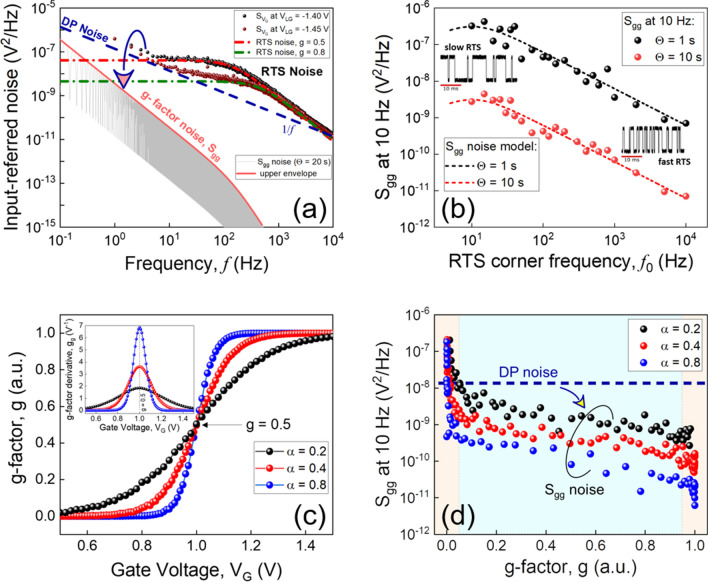
Noise suppression for the sensor based on the single-trap approach. (**a**) Input-referred noise of the 100 nm wide and 100 nm long Si NW FET measured at different liquid-gate voltages (indicated in the figure legend). The dash-dotted color lines denote the RTS components in the corresponding measured ${S}_{{V}_{G}}$ spectra. The dashed line represents DP noise calculated for the same liquid-gated Si NW FET. The arrow indicates the noise suppression. (**b**) ${S}_{gg}$ noise at 10 Hz calculated for the time windows $\Theta =1 s$ and $\Theta =10 s$, and plotted as a function of the RTS corner frequency when $g=0.5$. (**c**) Trap occupancy factor $g$ and its derivative ${g}_{g}$ (inset) calculated for the RTS noise simulated with different $\alpha$ (a ratio between gate capacitance ${C}_{G}$ and tunneling capacitance ${C}_{j}$ – see Equations (S1) and (S2) in SI). (**d**) Input referred g-factor noise ${S}_{gg}$ at 10 Hz and 10 s time window plotted as a function of $g$ for different conditions. Dashed blue line denotes here the DP noise level at 10 Hz calculated for the same transistor. The arrow indicates noise suppression below the thermal limit when considering RTS noise as a signal.

Figure 5a shows the input-referred voltage noise power spectral densities with Lorentzian fittings measured for the same 100 nm wide and 100 nm long liquid-gated Si NW FET demonstrating pronounced two-level RTS noise (see Fig. 4a). The dark blue dashed line represents the 1/*f* DP noise dependence calculated for the device using Eq. (2b) and considering *tg δ* = 3.8 × 10^–3^. As can be seen, the measured voltage noise is larger than the input-referred g-factor noise ${S}_{gg}$ calculated using Eqs. (10) and (11) with $g=0.5$, $\gamma =488 s^{-1}$ (as for the experimental data), and $\Theta =20 s$. Therefore, noise in the sensors exploiting the RTS phenomenon, in fact, can be suppressed when considering optimized conditions for calculation of g-factor. For this purpose, mainly three parameters need to be carefully considered: a time window $\Theta$, RTS frequency ${f}_{0}$, and a slope of g-factor dependence on the gate voltage applied.

The importance of the time window $\Theta$ on g-factor noise ${S}_{g}$ is shown in Fig. 4c. A large enough window that contains enough number of transition events (> 200)^38,39^ is needed for the meaningful statistical evaluation of $g$. However, the number of switching events between two levels within a given period of time also strongly depends on the RTS corner frequency ${f}_{0}$. The number of transitions over time $\Theta$ is higher for the high-frequency RTS than for the low-frequency RTS in the case of the same trap occupancy probability. Therefore, the g-factor can be evaluated with more accuracy for the fast RTS considering the same amount of time as for the slow (low-frequency) RTS process (see Fig. 5b).

The slope (steepness) of g-factor dependence on the gate voltage applied is another important parameter defining the efficiency of the single trap-phenomena for biosensing. The g-factor curves with different slopes are shown in Fig. 5c. For the sensors exploiting the RTS effect, the signal is the pronounced changes in RTS parameters (i.e. g-factor, capture time, etc.) induced by the depletion or accumulation of charge carriers in the silicon nanowire when the charged biomolecules are attached to its surface. Therefore, the sensitivity for the RTS-based sensors can be written as:

12 $${S}_{RTS}\approx \frac{\Delta g}{\Delta {V}_{G}}={g}_{g}$$

The sensitivity dependence on the g-factor slope (steepness) is proven also experimentally^22^. Moreover, according to Eq. (11), the input-referred g-factor noise also strongly depends on the slope of the g-factor curve. As can be seen in Fig. 5d, ${S}_{gg}$ noise can be, in fact, decreased by up to an order of magnitude due to the effect of the g-factor slope.

### Analysis of the signal-to-noise ratio for trap-based nanobiosensors

The signal-to-noise ratio is an important parameter for any sensor demonstrating its sensing capability. Therefore, this parameter needs to be carefully investigated for the nanotransistor sensors exploiting single-trap phenomena to optimize experimental conditions. Traditionally, for transistor-based biosensors monitoring threshold voltage shift as a signal, the signal-to-noise ratio can be defined as follows:

13 $$S/N=\frac{\delta {V}_{Th}}{\sqrt{{\int }_{f2}^{f1}{S}_{{V}_{G}}df}}$$

where ${S}_{{V}_{G}}$ is the equivalent input-referred voltage noise, and $\delta {V}_{Th}$ is a threshold voltage shift caused by the interaction of the target biomolecule with the sensing surface of the biosensor. The signal-to-noise ratio for nanobiosensors whose working principle is based on the single-trap phenomena can be determined similarly:

14 $$S/N=\frac{\delta {V}_{Th}}{\sqrt{{\int }_{f2}^{f1}{S}_{gg}df}}$$

**Figure 6:**
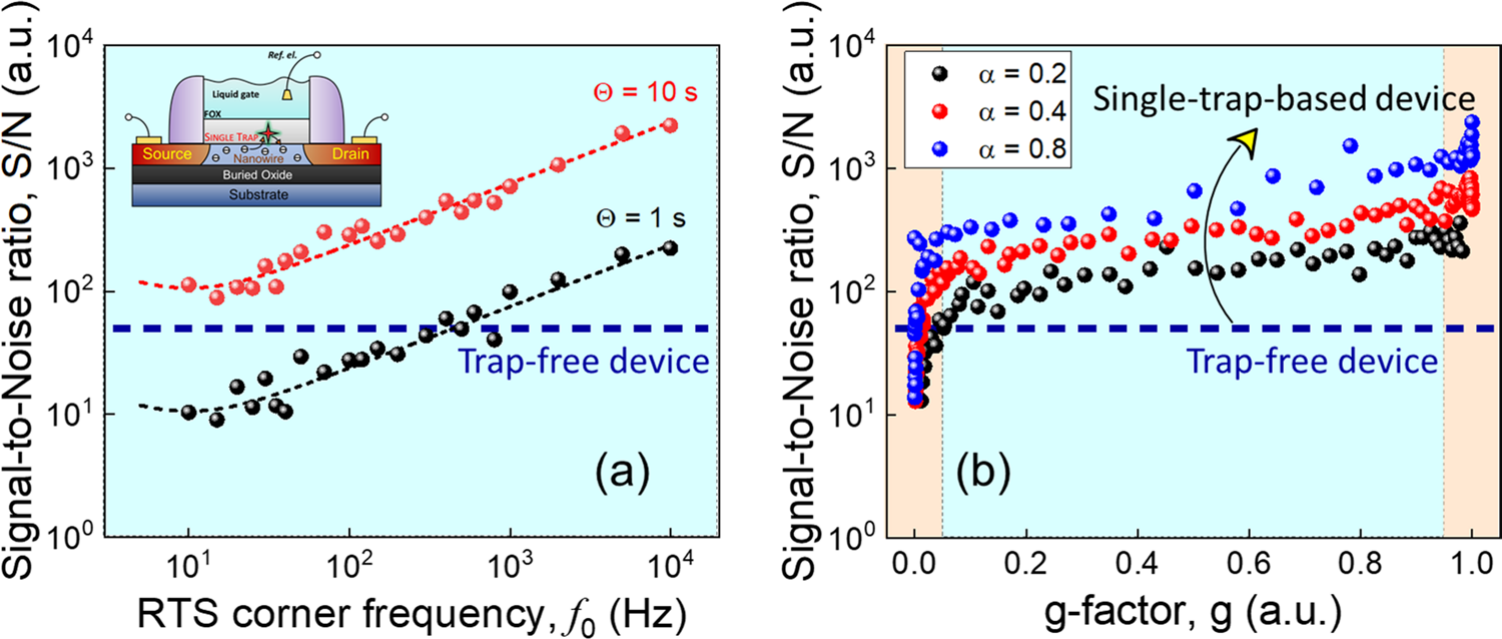
The signal-to-noise ratio of 5.9 mV signal estimated for the single-trap phenomena approach for different conditions. (**a**) The S/N ratio is calculated for the time windows $\Theta =1 s$ and $\Theta =10 s$, and plotted as a function of RTS corner frequency. (**b**) The S/N ratio vs. $g$ for different g-factor slopes and 10 s time window. The dashed line represents the S/N level calculated for the Si NW FET with DP voltage noise of 1.4 × 10^–8^ V^2^/Hz at 10 Hz. The arrow indicates the S/N ratio enhancement for the single-trap phenomena approach.

The S/N ratio calculated for RTS noise with different corner frequencies at $g=0.5$ is shown in Fig. 6a. A larger number of transition events due to the higher RTS rate ($\gamma ={\pi f}_{0}$) results in smaller ${S}_{gg}$ noise (see Fig. 5b) which leads to the increase of the S/N ratio. Figure 6b demonstrates the S/N ratio calculated for RTS phenomena with different g-factor slopes (see Fig. 5c). The dashed line reflects the S/N level for the trap-free device with the same gate capacitance as for one with the single trap demonstrating DP noise only. As a signal, we used the threshold voltage shift of 5.9 mV caused by 0.1 pH change in the gating solution when considering ideal ion-sensitive FET-based sensors. It can clearly be seen from Fig. 6a,b that under optimized conditions the S/N ratio can indeed substantially be increased even above the level expected for trap-free devices monitoring the threshold voltage shift as a signal.

## Discussion

The discrete number of traps in nanoscale devices offers a rich toolbox for optimizing the S/N ratio. In the case of the absence of traps, the dielectric loss of the gate oxide can be a tunable parameter in addition to the oxide thickness (see Fig. 2c,d). For single-trap phenomena, the RTS frequency is the main parameter as the best performance is obtained for a trap occupancy probability averaged over many events. The trap operation frequency is not easy to control, even though progress has been reported towards "on-demand" trap generation^23^. Instead, a simple way to increase the trap operation frequency is to consider a larger g-factor by tuning the gate bias (see Fig. 6b). This comes from the fact that ${\tau }_{e}$ is almost constant (see Fig. 4b) and therefore, at relatively high $g$, RTS corner frequency ${f}_{0}\approx 1/(2\pi {\tau }_{c})$. The alternative is to play with the slope of $g$ (Fig. 5d). In principle, the slope is only determined by the temperature (Fermi-distribution), and the trap depth (potential drop in the oxide), but in practice, punctual charges are very sensitive to correlation effects^19^ as often observed in electrochemical monolayers^12^. A gain in the S/N ratio is obtained in the case of "attractive" interactions.

It should be noted that in order to improve the S/N ratio for the single-trap phenomena approach applied for the biomolecular detection, the time window for the analysis of biomolecular signal should be optimized taking into account the parameters of the designed transducers and definite type of biological object under study. For example, in Ref.^32^ we measured and analyzed 40 s long RTS time traces to detect very low concentrations of target biomolecules. As a result, enhanced sensitivity was achieved and demonstrated for the single-trap phenomena approach. Our estimations, based on the equations in the present paper, show that in the case of a 40 s long time window, the g-factor noise is substantially lower compared to the measured RTS noise. This results in a considerably improved S/N ratio and demonstrates the validity of the approach.

From a more general perspective, single-trap phenomena can be considered as an SR effect when a white noise added to a signal enables better sensitivity and performance. As in biological systems, the white noise source is embedded. However, the particularity of single-trap phenomena is that the "discrete nature" of this white noise source is exploited (as in other single-electron devices^40^) as well as the fact that it is related to the physical parameters. Finally, one could argue that the best way to exploit single-trap phenomena would be to keep the signal digital, as it is an energy-efficient way of sensing and computing^41^, i.e. without requiring analog to digital converters.

## Summary and conclusion

The low-frequency noise plays an important role in any type of sensors determining their capability to detect small signals coming from the analyte. In this work, we have proposed and discussed the noise suppression techniques for FET-based nanosensors including the exploitation of RTS noise as a signal. We demonstrated that the signal-to-noise ratio can, in fact, considerably be increased for the single-trap phenomena approach. The results are very important for biosensing applications as well as for future nanotechnologies including the development of innovative charge-trap based memory devices and quantum computing systems.

## Supplementary information

Supplementary file1 (DOCX 435 kb)

## Data Availability

The data that support the findings of this study are available from the corresponding author upon reasonable request.
