# Supplementary material for: Noise suppression beyond the thermal limit with nanotransistor biosensors
Source: Sci Rep. 2020 Jul 29;10:12678. doi: 10.1038/s41598-020-69493-y (PMC7391715; doi:10.1038/s41598-020-69493-y)
Supplement: Supplementary file 1 — Supplementary file1 (DOCX 435 kb) [file 41598_2020_69493_MOESM1_ESM.docx]

**Noise Suppression Beyond the Thermal Limit with Nanotransistor Biosensors**

Yurii Kutovyi^1λ^, Ignacio Madrid^2λ^, Ihor Zadorozhnyi^1λ^, Nazarii Boichuk^1^, Soo Hyeon Kim^2^, Teruo Fujii^2^, Laurent Jalabert^2^, Andreas Offenhaeusser^1^, Svetlana Vitusevich^1^_,_ and Nicolas Clément^2*^

^1^ Bioelectronics (IBI-3), Forschungszentrum Jülich, 52425 Jülich, Germany

^2^ LIMMS-CNRS/IIS, Institute of Industrial Science, the University of Tokyo, 153–8505 Tokyo, Japan

^*^ Corresponding authors (emails: [nclement@iis.u-tokyo.ac.jp](mailto:nclement@iis.u-tokyo.ac.jp); [s.vitusevich@fz-juelich.de](mailto:s.vitusevich@fz-juelich.de))

**Note**: ^λ^ these authors have equally contributed to the work.

**Supporting Information**

1. **Si NW FET-based biosensors fabrication**

Si NW FET-based biosensors were fabricated on the basis of silicon-on-insulator (SOI) wafers with 50 nm thin <100>-oriented Si layer and 145 nm thick buried oxide layer. SOI wafers were purchased from SOITEC, France. Widths of designed and fabricated nanowires are in the range from 70 nm to 500 nm and lengths vary in the range from 100 nm to 4 µm. The detailed steps involved in the fabrication process of Si NW structures are described elsewhere^1^. Briefly, prior NW patterning, SOI wafers were covered with a 20 nm thick SiO_2_ layer using PECVD to serve as a layer for hard mask formation employing reactive ion etching. The NW pattern was defined using e-beam lithography and mesa structures were defined utilizing photolithography. After this, structures were transferred into the active silicon layer of SOI wafers using wet chemical etching in tetramethylammonium hydroxide (TMAH) solution. Afterward, ion implantation was performed with Boron or Arsenic atoms to create accumulation mode FETs (p^+^-p-p^+^) and inversion mode (n^+^-p-n^+^) transistor structures, correspondingly. Then, an 8-nm thin SiO_2_ layer was thermally grown on the NW structures to serve as the gate dielectric and protect the conductive channel of transistor-based biosensors from the liquid environment. The metallization process was then performed by sputtering of a metal stack consisting of 5 nm TiN and 200 nm of Al followed by the lift-off patterning and annealing process. To protect metal leads against the liquid environment, the structures were passivated with a polyimide layer. The access of the liquid solution to Si NWs was provided by patterning the passivation layer with photolithography. After the fabrication process wafers were cut into chips which were further encapsulated and used for the biosensing experiments. All the fabrication steps were performed at the Helmholz Nano Facility (HNF) of Forschungszentrum Jülich.

1. **Characterization of the fabricated Si NW FET structures**
   1. **Current-voltage characterization**

Silicon nanowires with lengths in the range from 100 nm to 4 µm and widths from 70 nm to 500 nm were investigated from the viewpoint of scalability, device performance as well as noise properties. Transfer curves measured for p^+^-p-p^+^ and n^+^-p-n^+^ structures with different lengths and width of 100 nm are shown in Fig.S1(a). Characteristics were measured in linear operation regime at the absolute value of drain-source voltage V_DS_ = 100mV.


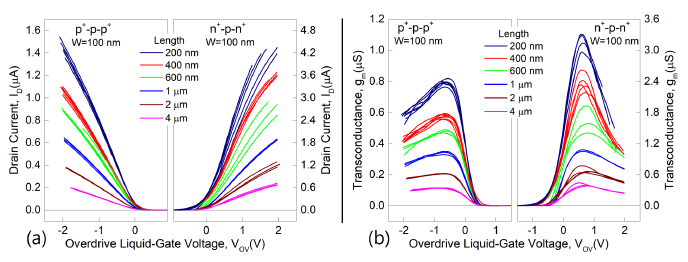


**Figure S1.** (a) Transfer curves measured for p^+^-p-p^+^ and n^+^-p-n^+^ structures with different lengths. (b) Transconductance recalculated as a derivative from the transfer curves, shown in (a).

Si NW FET structures demonstrate good scalability with the length of the nanowire. This is also valid for transconductance (Fig.S1(b)), recalculated as a derivative of transfer curves. It should be noted that transconductance maximum is also around three times larger for n^+^-p-n^+^ structures in comparison to p^+^-p-p^+^ analogs.

- 1. **Noise characterization**

Noise characterization of silicon nanowire devices was performed using the noise measurement setup described in detail elsewhere^1–3^. Noise characteristics were measured in different operation modes for the Si NW FET structures with various nanowire widths and lengths. At low frequencies, noise spectra of measured nanowires demonstrated the 1/f flicker noise behavior, which is dominant for major Si NW chips under investigation. The generation-recombination (GR) noise components appearing in the noise spectra for nanowires with small characteristic sizes correspond to single-electron events resulting in random telegraph signal fluctuations of the current through the channel. The values of the current noise power spectral density taken at 10 Hz as a function of the overdrive gate voltage are plotted in Fig.S2(a) and S2(b) for p^+^-p-p^+^ and n^+^-p-n^+^ structures with different widths, respectively.


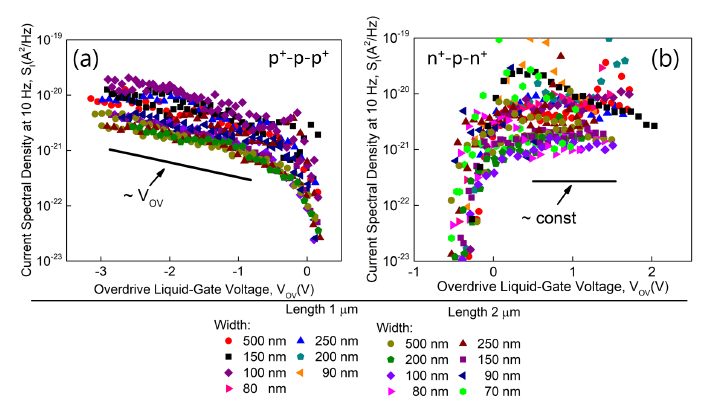


**Figure S2.** Current noise spectral density measured for the nanowires of different widths in the range from 70 nm to 500 nm and lengths of 1 and 2 microns for (a) p^+^-p-p^+^ and (b) n^+^-p-n^+^ liquid-gated structures.

The normalized current spectral density at 10 Hz is shown in Fig.S3. The p-type devices demonstrate I_D_^-1^ behavior (Fig.S3(a)) while n-type – I_D_^-2^ (Fig.S3(b)). Such variation can be explained by different origins of noise for n- and p-type structures.


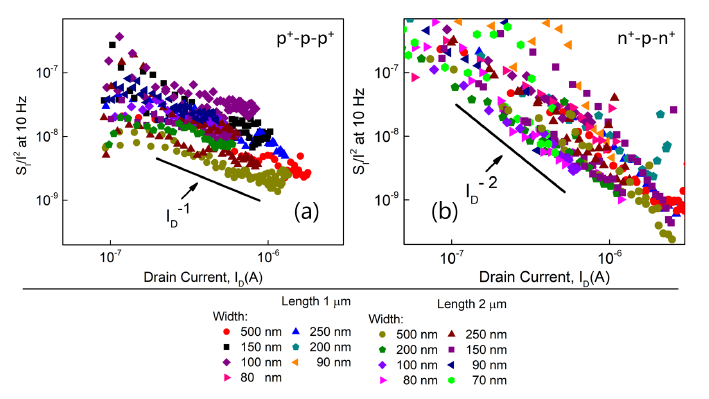


**Figure S3.** Normalized current noise spectral density measured on the nanowires of different widths in the range from 70 nm to 500 nm and lengths of 1 and 2 microns for (a) p^+^-p-p^+^ and (b) n^+^-p-n^+^ configurations.

Input-referred noise as a function of the gate area is shown in Fig.S4(a) and 4S(b) for p-type and n-type devices, respectively. As it is predicted by the noise model (see Equation (1b) in the manuscript) input-referred noise for both n- and p-type FETs is inversely proportional to the gate area. As it is shown in Fig.S4 both device types follow predicted behavior.


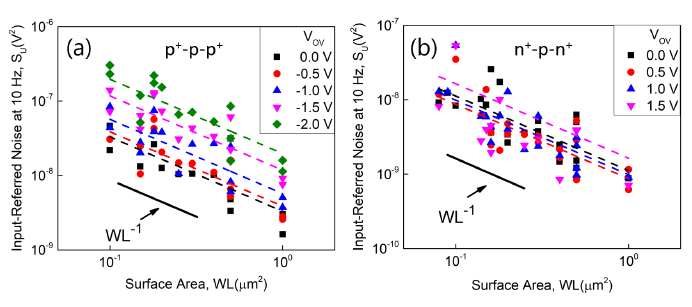


**Figure S4.** The input-referred noise as a function of gate surface area for (a) ) p^+^-p-p^+^ and (b) n^+^-p-n^+^ structures at different overdrive liquid-gate voltages.

1. **Random telegraph signal noise simulation procedure**

To evaluate the probabilities of electrons to be captured or emitted from the trap we utilized a method previously described in ref^4^. The capture and emission rates were defined using the following formulas:

|  | $R_{c}=\frac{1}{\tau_{c}}=R_{coeff}* dgn*\exp\left[ -\frac{q}{kT} \gamma(E_{trap}- \alpha V_{g}) \right]$ | (1) |
| --- | --- | --- |

|  | $R_{e}=\frac{1}{\tau_{e}}=R_{coeff}* dgn*\exp\left[ \frac{q}{kT} (1-\gamma) (E_{trap}- \alpha V_{g}) \right]$ | (2) |
| --- | --- | --- |

where $R_{coeff}$ is a coefficient defining rate when $g=0.5$, $dgn$ denotes a degeneracy factor (set to 1 for all simulations), $q$ is the elementary charge, $\gamma$ is the charge transfer coefficient (1 if $\tau_{e}$ doesn’t depend on $V_{g}$), $E_{trap}$ is the energy of a trap, $V_{g}$ is applied gate voltage, $\alpha$ is the ration between the gate capacitance and tunneling capacitance^5^ $C_{j}$ – capacitance between the channel and the trap. Therefore, we assume that $\alpha$ is the ratio between the trap depth $d_{trap}$ and the dielectric thickness$t_{ox}$.

The probability of the electron transferring (capture or emission) at time *t* since the last transition was calculated using formulas:

|  | $P_{c,e}=1-Exp[-\frac{t}{\tau_{c, e}}]$ | (3) |
| --- | --- | --- |

1. **Autocorrelation function of g-factor**


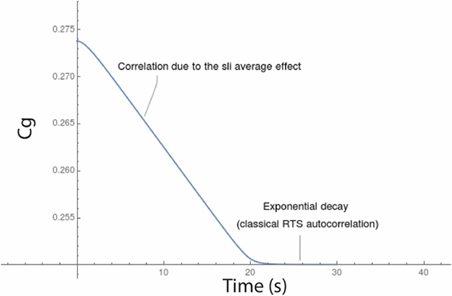


**Figure S5.** The correlation function of g-factor as a function of the averaging time window. Two regimes are observed that are related to the averaging filter and the stochastic charge transfer, respectively.

**References**

1. Zadorozhnyi, I. Nanowire Transistor Structures for Advanced Biosensing. *Doctoral thesis* (Forschungszentrum Juelich and TU Dortmund University, 2019). doi:dx.doi.org/10.17877/DE290R-20179

2. Petrychuk, M. *et al.* Noise spectroscopy to study the 1D electron transport properties in InAs nanowires. *Nanotechnology* **30**, 1–9 (2019).

3. Kutovyi, Y. *et al.* Liquid-Gated Two-Layer Silicon Nanowire FETs: Evidence of Controlling Single-Trap Dynamic Processes. *Nano Lett.* **18**, 7305–7313 (2018).

4. Huang, K. C. & White, R. J. Random walk on a leash: A simple single-molecule diffusion model for surface-tethered redox molecules with flexible linkers. *J. Am. Chem. Soc.* **135**, 12808–12817 (2013).

5. Clément, N., Nishiguchi, K., Fujiwara, A. & Vuillaume, D. One-by-one trap activation in silicon nanowire transistors. *Nat. Commun.* **1**, 1–8 (2010).
